# Early recognition of anorexia through patient-generated assessment predicts survival in patients with oesophagogastric cancer

**DOI:** 10.1371/journal.pone.0224540

**Published:** 2019-11-27

**Authors:** Marc Abraham, Zoe Kordatou, Jorge Barriuso, Angela Lamarca, Jamie M. J. Weaver, Claudia Cipriano, George Papaxoinis, Alison Backen, Wasat Mansoor

**Affiliations:** 1 Department of Nutrition & Dietetics, The Christie NHS Foundation Trust, Manchester, United Kingdom; 2 Department of Medical Oncology, The Christie NHS Foundation Trust, Manchester, United Kingdom; 3 Division of Cancer Sciences, School of Medical Sciences, Faculty of Biology, Medicine and Health, University of Manchester, Manchester, United Kingdom; Universidade do Algarve Departamento de Ciencias Biomedicas e Medicina, PORTUGAL

## Abstract

Cancer cachexia is common in patients with oesophagogastric cancer (OG) and is linked to overall survival (OS). One of the key components of cachexia is anorexia; it is not known whether anorexia impacts on OS and there is no method of routine screening in current practice. Diagnosis relies on patients describing the symptoms, clinicians diagnosing anorexia and acting upon it. Patients with oesophageal/gastroesophageal junction or gastric cancer were assessed using the Functional Assessment of Anorexia Cachexia Therapy Anorexia/Cachexia Subscale (FAACT A/CS). FAACT A/CS includes 12 questions validated previously to diagnose anorexia in patients with cancer. Of the 182 patients included, 69% scored ≤37/48 and were considered to be anorexic; FAACT A/CS was a better predictor of OS in metastatic patients than body mass index or weight loss in the six months prior to cancer diagnosis. The median OS of patients with FAACT A/CS scores of >37 was longer than patients with scores of ≤37 (19.3 months vs 6.7 months, Hazard Ratio [HR] 2.9, 95% Confidence Interval [CI] 1.4–6.0, p<0.0001). Patients with performance status (PS) 0–2 and FAACT A/CS >37 had substantially longer OS than those with PS 0–2 and FAACT A/CS ≤37 (18.7 months vs 7.9 months, HR 2.5 (95% CI 1.2–5.1, P<0.0001). The FAACT A/CS questionnaire allows clinicians to identify patients with anorexia who may benefit from early nutrition interventions. Importantly, this is the first study to show the association between anorexia and survival in patients with metastatic OG cancers. This will form the basis of future interventional studies to improve patient outcomes.

## Introduction

Cancer cachexia is a condition defined by loss of skeletal muscle mass. It is characterised by negative calorie and nitrogen balance, driven by a combination of reduced food intake and abnormal metabolism [1]. This complex interaction results in weight loss and reduces both quality of life and survival outcomes [2].

There are defined stages of cancer cachexia; pre-cachexia, cachexia and refractory cachexia. Not all patients may go through these stages but there is currently no biomarker to identify pre-cachectic patients who may benefit from early management interventions. The refractory stage can be considered as cachexia with very poor prognosis, as it is the cancer disease that defines this stage [1,3].

Cachexia is further compounded in patients with oesophagogastric (OG) cancers because of disease-related issues such as oesophageal or gastric outlet obstruction due to the physical location of the primary cancer, and reduced food intake and nutrient absorption which can be caused by mouth ulcers, altered bowel habit, vomiting, pain, the impact of systemic antineoplastic therapy, radiotherapy or malabsorption [4]. Cachexia in patients with OG cancers may be substantially underdiagnosed due to poor methods of detection and the lack of motivation to investigate and manage these complex conditions (in addition to the cancer diagnosis).

Assessment for cachexia should include investigations into anorexia, catabolic drive, muscle mass and strength, and functional and psychosocial impairment [1]. Components of cachexia that can be diagnosed objectively include weight loss and sarcopenia (low muscle mass). Diagnostic criteria for cachexia include weight loss of greater than 5% in the previous 6 months, or body mass index (BMI) less than 20 kg/m^2^ but with more than 2% weight loss, or sarcopenia with weight loss of greater than 2% in individuals. Weight loss can be accurately measured by standardising the procedures methodology but there are limitations with interpreting reported weight changes pre-diagnosis as it is difficult to ascertain the time at which the pre-cancerous individual develops cancer and patient recall bias is also possible [5]. BMI may not be helpful in predicting survival outcome in the metastatic OG cancer cohort [6] and although sarcopenia can be measured using computed tomography or dual-energy X-ray absorptiometry, the lack of standardisation of methods for assessing and reporting body composition in this patient group limits assessment [7,8]. However, these assessment methods are not easily available in cancer clinics [3].

Given the complex and multifactorial nature of cachexia, each component may contribute differing extents to a patient’s deterioration in quality of life and overall survival; importantly they require different approaches to management making it important to diagnose and consider each of these factors separately. Therefore, it has been proposed that a multimodal management approach is indicated [9].

Anorexia, the lack of desire to eat resulting in reduced food intake, is a key feature of cachexia, even in the pre-cachectic state. It is very common in patients with OG cancer, is independently associated with a poorer quality of life [10] and has been shown to be a major source of distress for patients and their carers [11]. Although anorexia in cancer patients is widely reported in the literature, identifying patients at risk remains a challenge due to the subjective nature of a patient describing anorexia in consultation. There is no standardisation for physicians to capture the information reported by patients, measure the severity or record the key information. As a result, the effects of anorexia on patient outcomes, including survival and quality of life, remain inconsistently reported and poorly understood. Even when it is identified, it is often seen as low priority in clinical practice.

The Functional Assessment of Anorexia Cachexia Therapy Anorexia/Cachexia Subscale (FAACT A/CS) [12] identifies patients with anorexia; the questionnaire has a total possible score of 48 and a cut-off score of ≤37 has been validated to diagnose anorexia in patients with cancer [13].

In this study, we hypothesised that anorexia, diagnosed based on the FAACT A/CS, was a more effective tool than either BMI or weight change pre-diagnosis in providing information about the process of deterioration in nutritional pathology and association with survival outcomes in patients with OG cancer.

## Methods

Consecutive patients with newly diagnosed oesophageal, gastroesophageal junction or gastric adenocarcinoma (of all stages) who attended The Christie Hospital NHS Foundation Trust between September 2016 and December 2017 were recruited to the study. This was a fact-finding study to generate clinical hypotheses for future studies; there were no power or sample size calculations because prior to this study, there were no data to perform these calculations accurately.

Baseline characteristics, such as number and location of metastases, age, gender, Eastern Cooperative Group Performance Status (ECOG PS), were collected at the time of first attendance at The Christie NHS Foundation Trust. All patient data including survival outcome were retrospectively analysed and de-identified before the authors gained access. All patients completed the FAACT A/CS questionnaire prior to any anti-cancer treatment. The FAACT A/CS questionnaire was devised in 2000 [12] and consists of twelve questions, which each have five possible multiple choice answers (not at all, little bit, somewhat, quite a bit, very much). This is shown in Table 1.

**Table 1:** The FAACT A/CS questionnaire. Reproduced with permission from Functional Assessment of Chronic Illness Therapy (FACIT). FAACT A/CS; Functional Assessment of Anorexia Cachexia Therapy Anorexia/Cachexia Subscale.

The patient’s response applies to the last week		Not at all	A little bit	Somewhat	Quite a bit	Very much
**1**	I have a good appetite	0	1	2	3	4
**2**	The amount I eat is sufficient for my needs	0	1	2	3	4
**3**	I am worried about my weight	4	3	2	1	0
**4**	Most food tastes unpleasant to me	4	3	2	1	0
**5**	I am concerned about how thin I look	4	3	2	1	0
**6**	My interest in food drops as soon as I try to eat	4	3	2	1	0
**7**	I have difficulty eating rich or ‘heavy’ foods	4	3	2	1	0
**8**	My family or friends are pressuring me to eat	4	3	2	1	0
**9**	I have been vomiting	4	3	2	1	0
**10**	When I eat, I seem to get full quickly	4	3	2	1	0
**11**	I have pain in my stomach area	4	3	2	1	0
**12**	My general health is improving	0	1	2	3	4

The scoring system awards points from 0 to 4 for each question and is arranged so that the higher values equate to healthier scenarios. Each patient’s response applies to the previous week and a total score of up to 48 is possible. A total score cut-off of ≤37 has been validated to diagnose anorexia in patients with cancer [13].

All patients had their height and weight measured. Body Mass Index (BMI), O’Rourke dysphagia score [14] and weight change over the 6 months prior to diagnosis (from patient recollection) were also recorded. The O’Rourke dysphagia score was assessed and scored as follows; normal eating (1), eats soft food only (2), eats purified food only (3), drinks liquids only (4) and no swallowing at all (5).

For all patients with metastatic disease the following additional information was collected; number and location of metastatic sites, whether the patient received chemotherapy, their ECOGPS and survival outcomes.

A BMI-adjusted weight loss grading system for patients with cancer cachexia has been devised which combines weight loss with BMI to produce a grade from 0 to 4; this grading system was used exactly as intended (higher weight loss equates to a worse prognosis) [15].

Associations between independent categorical variables were examined by chi-square test. Continuous variables were described by mean value and standard deviation (SD). Mean values were compared by type 2, 2-tailed, t-test. Survival analysis was performed by using the Kaplan-Meier estimator method, log rank test and univariate Cox regression. Survival data were last updated on 02 August 2018; living patients were censored on that date. Multivariable Cox proportional hazard models were used to examine variables independently associated with survival; this was performed separately for patients who did and did not receive treatment for their cancer. Interaction between treatment and the FAACT A/CS questionnaire was explored in the Cox regression model. Two-tailed p-values (p) of <0.05 were considered significant. SPSS version 19 (IBM, New York, USA) and GraphPad Prism version 7 (GraphPad Software Inc, California, USA) were used for statistical analyses.

### Ethics approval and consent to participate

Approval to conduct this study was formally granted by the Clinical Audit Committee of The Christie NHS Foundation Trust on 26 April 2017 (reference 17/1944). All participants granted informed oral consent to participate in a single FAACT A/CS questionnaire and to having minimal anonymised clinical collected. This study was performed in accordance with the Declaration of Helsinki.

## Results

**Fig 1:**
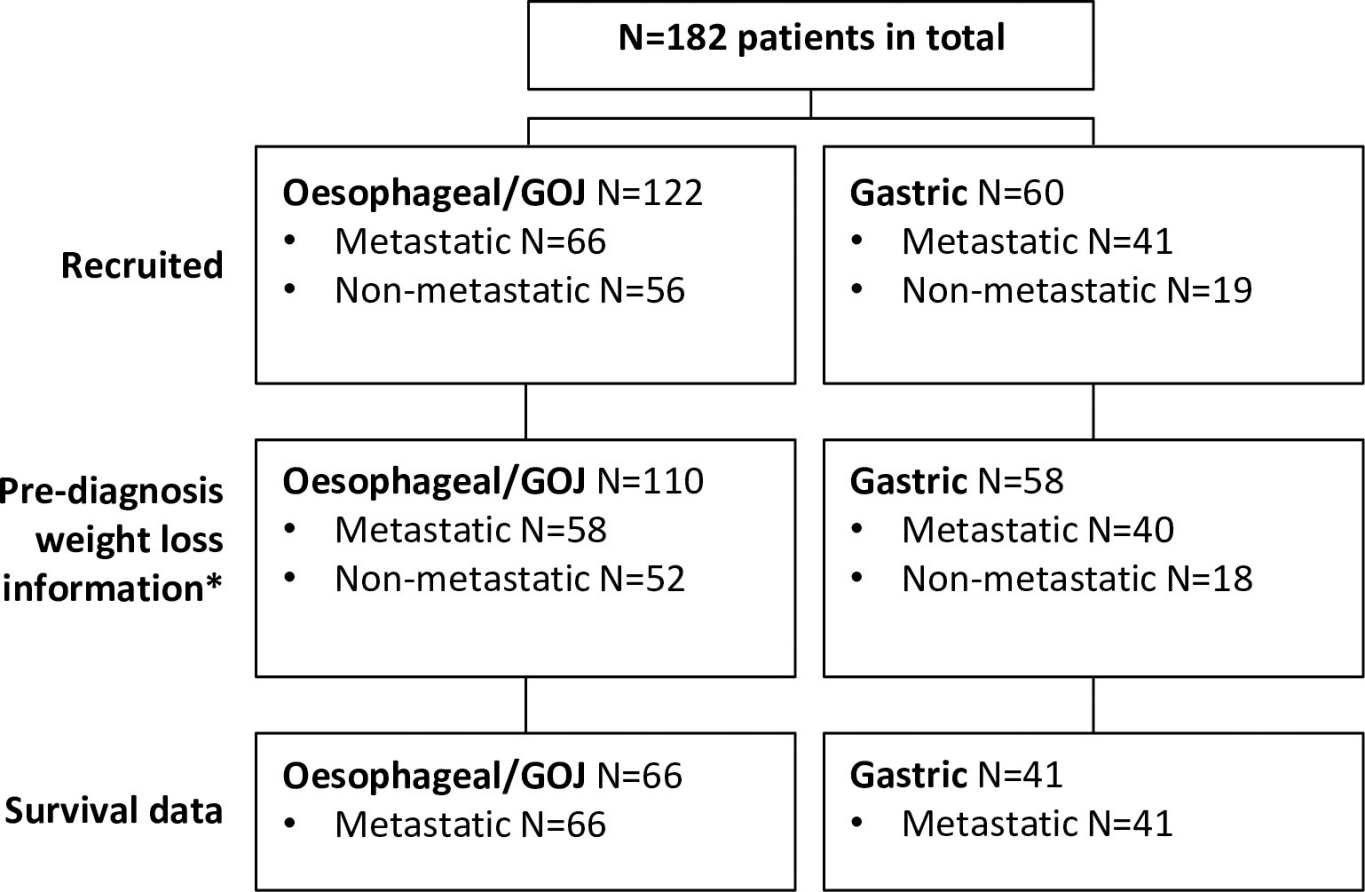
Patient overview. This is an overview of patients included in this study. *N = 14 patients were categorised as ‘unclear’ weight loss over the previous 6 months, so have been removed from all evaluations involving weight loss. Survival data was available for patients with metastatic cancer. **GOJ;** gastroesophageal junction cancer, **N;** number of patients.

A total of 182 consecutive patients with newly-diagnosed oesophageal/GOJ (N = 122) or gastric adenocarcinoma (N = 60) were recruited. **Fig 1** shows an overview of the patients included in this study. **Table 2** summarises the characteristics of the patients included in this study. Mean age was 65.7 years, median 67 years (range 37–85). The number of metastatic sites was considered; 48% of patients had just one site, 37% had two, 18% had three and 3% had four sites (maximum number of sites). Patients with oesophageal/GOJ cancer more frequently had liver metastases than those with gastric cancer (33 v 12 patients) and conversely, patients with oesophageal/GOJ cancer had fewer peritoneal metastases than those with gastric cancer (6 v 16 patients).

**Table 2:** Patient characteristics of all patients in this study.

	Metric	All patients	Oesophageal/GOJ patients	Gastric patients
**All patients in this study**	**Number of patients** N, (%)	182 (100%)	122 (67%)	60 (33%)
	**Height** Mean m, (range)	1.70 (1.42–1.9)	1.72 (1.42–1.94)	1.68 (1.50–1.84)
	**Weight** Mean kg, (range)	76.3 (39–170)	78.1 (39–135)	72.7 (41–170)
	**Body Mass Index** Mean kg/m^2^, (range)	26.1 (15–52)	26.4 (15–52)	25.6 (16–52)
	**O’Rourke** **dysphagia score** (N)	1 (96), 2 (49), 3 (24), 4 (12), 5 (1)	1 (42), 2 (47), 3 (21), 4 (12), 5 (0)	1 (54), 2 (2), 3 (3), 4 (0), 5 (1)
	**Pre-diagnosis weight change**^**1**^ mean %, (range)	-5.7 (-51–0)	-5.4 (-26–0)	-6.4 (-51–0)
	**FAACT A C/S total** Mean score, (range)	30 (3–48)	31 (4–48)	27 (3–48)
**Only patients with metastatic disease**	**Number of patients with metastases** N, (%)	107 (59%)	66 (54%)	41 (68%)
	**Age** Mean years (range)	65.7 (37–85)	66.9 (47–85)	65.4 (37–82)
	**Gender** N male, (%)	80 (75%)	56 (85%)	24 (59%)
	**Number of metastatic sites**^**2**^ 0–4 (N)	1(48), 2(37), 3(18), 4(3)	1(29), 2(21), 3(16), 4(0)	1(19), 2(16), 3(2), 4(3)
	**Liver metastases** N, (%)	45 (43)	33 (50)	12 (30)
	**Peritoneal metastases** N, (%)	22 (21)	6 (9)	16 (40)
	**Other metastases**^**3**^ N, (%)	88 (83)	54 (82)	34 (85)
	**Cancer treatment** N yes, (%)	77 (72%)	47 (71%)	30 (73%)
	**ECOG Performance Status 0–3** (N)	0 (25), 1 (51), 2 (20), 3 (11)	0 (19), 1 (30), 2 (11), 3 (6)	0 (6), 1 (21), 2 (9), 3 (5)
	**Overall survival** Mean months, (range)	8.8 (0.7–23.0)	9.4 (0.7–21.5)	7.9 (1.0–23.0)

This is an overview of the characteristics of the patients included in this study.

^1^Patients were asked to provide information about how much their weight had changed in the 6 months before their cancer diagnosis N = 14 patients were unclear about weight change (N = 12 oesophageal/GOJ [N = 8 metastatic, N = 4 non-metastatic], N = 2 Gastric [N = 1 metastatic, N = 1 non-metastatic]); these 14 patients were removed from all evaluations of weight change.

^2^N = 1 patient with gastric cancer did not have the number of metastatic sites documented.

^3^Other metastatic sites included adrenal, bone, lung, nodes, omentum, ovary, renal, retrothyroid, pleura and skin.

**ECOG;** Eastern Cooperative Group, **GOJ;** gastroesophageal junction cancer, **FAACT A/CS;** Functional Assessment of Anorexia Cachexia Therapy Anorexia/Cachexia Subscale, **N;** number of patients.

## Association of basic tumour characteristics with somatometric parameters

There was no difference in mean body weight (P = 0.06) or BMI between cancer types (P = 0.34).

**Fig 2:**
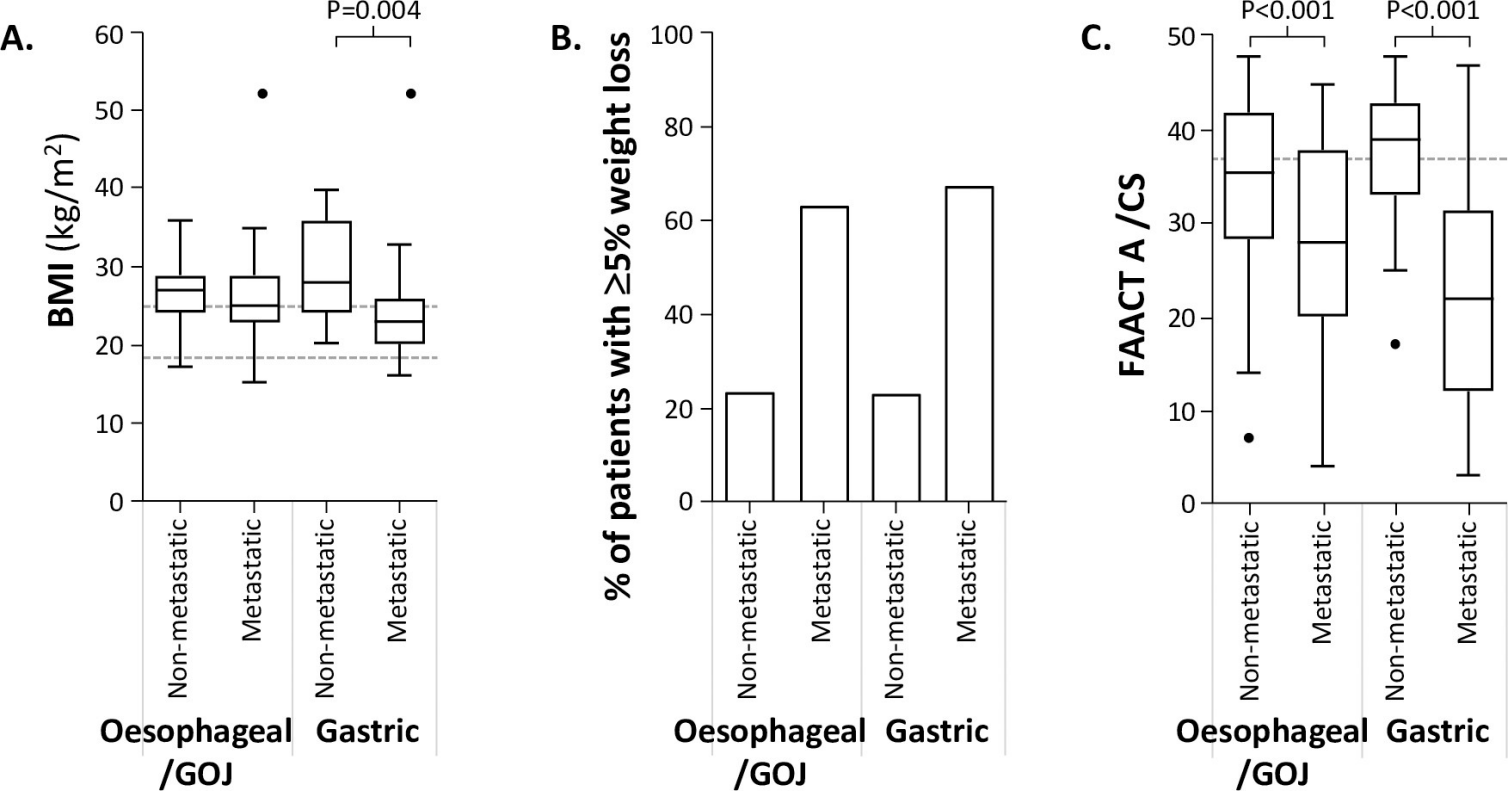
Evaluation of BMI, pre-diagnosis weight loss and FAACT A/CS. **A.** BMI levels; box and whisker plots show the median, upper and lower quartiles and maximum/minimum (excluding outliers), the dotted lines indicate the upper and lower limits of ‘normal’ BMI, **B.** Percent of patients with ≥5% weight loss, **C**. Total FAACT A/CS levels; box and whisker plots show the median, upper and lower quartiles and maximum/minimum (excluding outliers), the dotted line indicates the validated ≤37 threshold below which patients with cancer are considered to be anorexic.

**Fig 2A** shows that there was no significant difference in BMI values between metastatic and non-metastatic oesophageal/GOJ cancer patients [mean BMI 25.9 (SD 5.5) v BMI 26.9 (SD 4.2) respectively, P = 0.27]. However, among patients with gastric cancer, those with metastatic disease had a significantly lower BMI than those with metastatic disease [mean BMI 23.9 (SD 6.0) v BMI 29.7 (SD 6.3) respectively, P = 0.004].

N = 14 patients were categorised as ‘unclear’ weight loss over the previous 6 months, so have been removed from all evaluations involving weight loss. P values indicated are t-test (type 2, 2-tailed). N = 14 patients were categorised as ‘unclear’ weight loss over the previous 6 months, so have been removed from all evaluations involving weight loss. **BMI;** body mass index, **FAACT A/CS;** Functional Assessment of Anorexia Cachexia Therapy Anorexia/Cachexia Subscale, **GOJ;** gastroesophageal junction cancer.

## Association of tumour characteristics with dysphagia, weight loss and anorexia

As expected, O'Rourke score was lower (better) in patients with gastric cancer (mean score 1.2, SD 0.7) than in patients with oesophageal/GOJ cancer (mean score 2.0, SD 1.0, P<0.0001). No difference was observed in O'Rourke scores between patients with metastases (mean score 1.7, SD 0.9) and without metastases (mean score 1.8, SD 1.0, P = 0.7).

The majority of patients reported weight loss, whereas nobody reported having gained weight. Fourteen patients (7.7% of the total) were unclear about whether their weight had changed, so they have been excluded from all evaluations involving weight loss. There was no difference in percentage weight decrease in the previous 6 months between patients with oesophageal/GOJ cancer and gastric cancer [mean -5.4% loss (SD 5.8%) v mean -6.5% loss (SD 7.8%), respectively, P = 0.38]. However patients with metastatic disease had experienced more weight loss than patients with localised disease. This is summarised in **Fig 2B**; more than twice as many patients with metastatic cancer had ≥5% weight loss in the previous 6 months in both oesophageal/GOJ and gastric cancer.

Patients with oesophageal/GOJ cancer had higher (better) scores in the FAACT A/CS questionnaire than patients with gastric cancer [FAACT A/CS mean 31 (SD 11) v mean 27 (SD 13), respectively, P = 0.03]. **Fig 2C** shows that in patients with oesophageal/GOJ and gastric cancer, patients with metastatic disease scored substantially lower than those with localised disease, p<0.0001.

**Fig 3:**
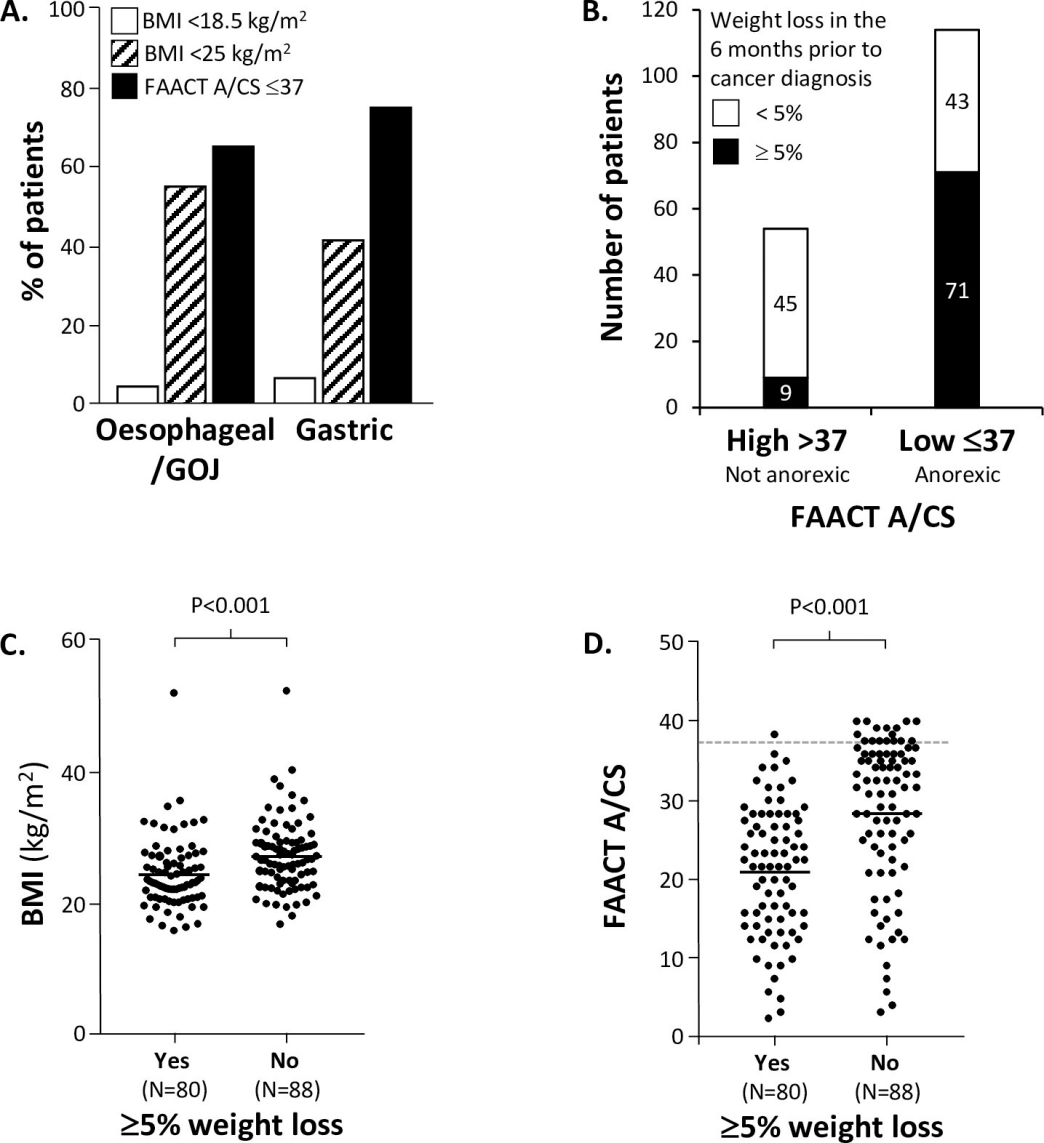
Evaluation of BMI, pre-diagnosis weight loss and FAACT A/CS in all patients. **A.** A comparison of the patients who are considered to be under-weight by using BMI (using the lower and upper thresholds of the normal range) and FAACT A/CS. **B.** Relationship between FAACT A/CS score and weight loss; 17% of people with high FAACT scores, had lost ≥5% weight and 62% of people with low FAACT scores, had lost ≥5% weight. **C.** Difference in BMI levels in patients who did and did not have ≥5% weight loss and **D.** Differences in FAACT A/CS in the same patient population, the dotted line indicates the validated ≤37 threshold below which patients with cancer are considered to be anorexic. N = 14 patients were categorised as ‘unclear’ weight loss over the previous 6 months, so have been removed from all evaluations involving weight loss. P values indicated are t-test (type 2, 2-tailed). N = 14 patients were categorised as ‘unclear’ weight loss over the previous 6 months, so have been removed from all evaluations involving weight loss. **BMI;** body mass index, **FAACT A/CS;** Functional Assessment of Anorexia Cachexia Therapy Anorexia/Cachexia Subscale, **GOJ;** gastroesophageal junction cancer.

**Fig 3A** compares the proportions of patients considered to be under-weight (using different methods); 5% of all patients had a BMI of <18.5 kg/m^2^ (lower limit of normal), 51% of all patients had a BMI of ≤25 kg/m^2^ (upper limit of normal) and 49% of all patients had a BMI of >25 kg/m^2^, but 69% of all patients scored ≤37 on the FAACT A/CS and are considered to be anorexic.

**Fig 3B** examines the relationship between FAACT A/CS score and weight loss; only 17% of patients with high FAACT scores (>37), who are not considered to be anorexic, had lost ≥5% weight, while 62% of patients with low FAACT scores (≤37) who are considered to be anorexic, had lost ≥5% weight. Although there was a strong association between anorexia and weight loss, we demonstrated that these to parameters are not equivalent.

**Fig 3C** examines the ≥5% weight loss threshold and shows that the difference between the mean BMI values of patients who did and did not lose ≥5% weight in the six month prior to cancer diagnosis. Although the values are statistically-significantly different [means of 24.0 (SD 5.0) vs. 28.7 kg/m^2^ (SD 5.0), respectively, P<0.001), because the means are similar, this is unlikely to be a useful discriminator when considering each patient in clinic. The difference between the mean FAACT A/CS values (**Fig 3D**) of the two populations [means of 24.0 (SD 5.0) vs. 37.3 kg/m^2^ (SD 8.8), respectively, p<0.001] is more likely to be a useful discriminator in clinic. It is interesting to note that only 9% of patients who had experienced ≥5% weight loss had FAACT A/CS scores of >37, but 62% of patients who had not experienced ≥5% weight loss had FAACT A/CS scores of >37.

## Analysis of prognostic factors

Survival data was available for patients with metastatic cancer (median OS had not been reached in the non-metastatic group). The survival outcomes for the N = 107 patients with metastatic disease were calculated; the bottom-part of **Table 2** summarises the additional characteristics collected from these patients.

There was no difference in survival outcomes for young (below the median age) or older (above the median age) patients with metastatic disease (8.0 months v 9.0 months, respectively, P = 0.73).

As expected, patients with metastatic disease who received treatment for their cancer (N = 77), had longer OS than those who did not (N = 30). A similar percentage of patients with oesophageal/GOJ and gastric cancers received treatment. Median OS of patients who received treatment was 12.2 months, while in those without treatment it was 3.3 months (Hazard Ratio (HR) 3.7, 95% confidence interval (CI) 2.4–5.9, P<0.001).

**Fig 4:**
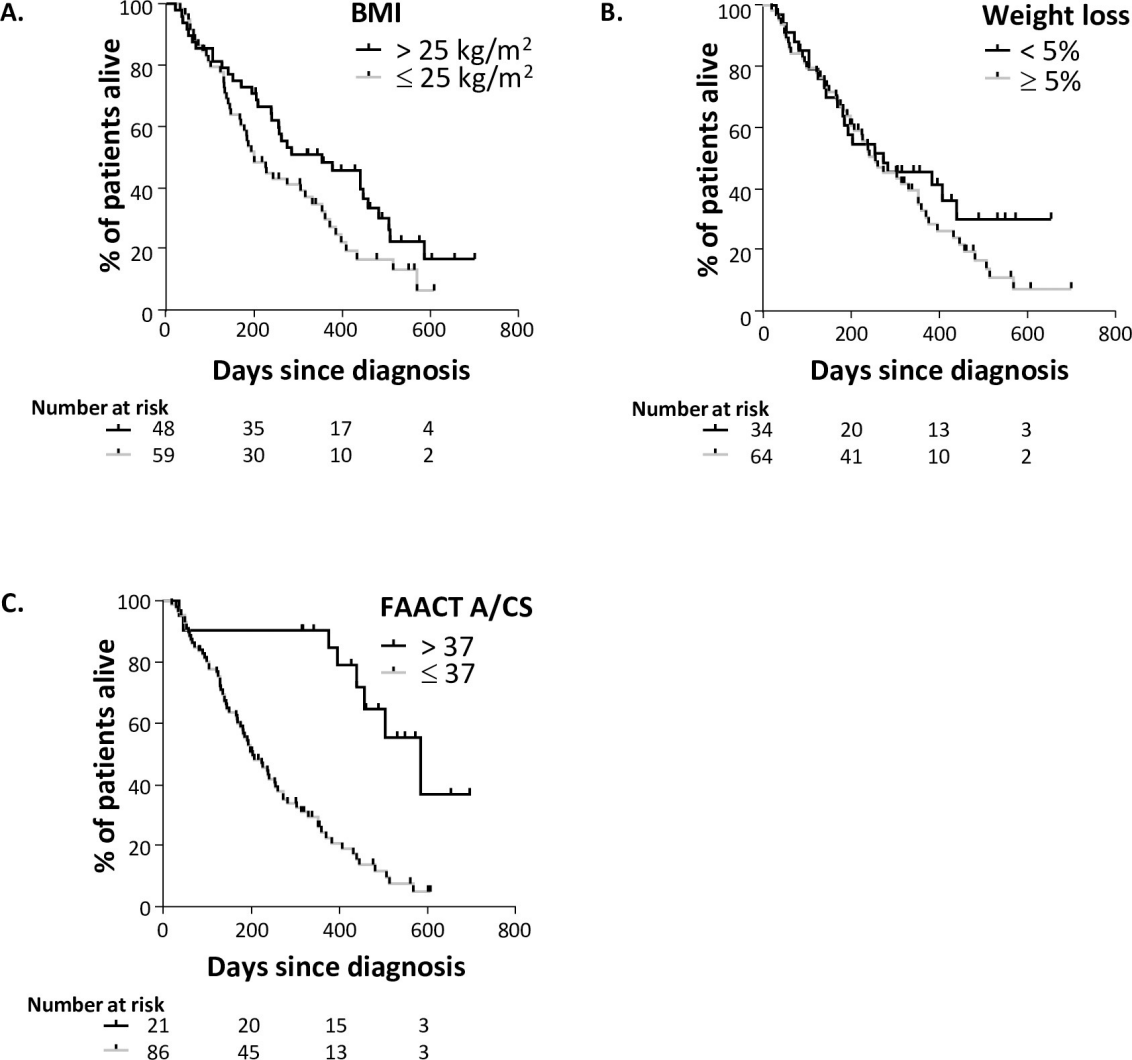
Overall survival outcomes in N = 107 patients with metastatic cancer. **A.** Survival outcomes for patients divided by BMI (using the upper limit of normal as a cut-point), the median OS for patients with higher BMI (> 25 kg/m^2^) was longer (11.6 months) than patients with lower BMI (6.5 months), log-rank (Mantel-Cox) Chi square = 3.9, HR 1.8, 95% CI 1.1–2.8 P = 0.05. **B.** Survival outcomes for patients divided by weight loss; the median OS for patients who had lost less than 5% weight loss in the six months prior to their cancer diagnosis was similar (9.0 months) than patients with who had experienced ≥5% weight loss (8.4 days), log-rank (Mantel-Cox) Chi square = 1.3, HR 0.93, 95% CI 0.56–1.56 P = 0.26. **C.** Survival outcomes for patients divided by FAACT A/CS; the median OS for patients with FAACT A/CS scores of >37 was longer (19.3 months) than patients with scored of ≤37 (6.7 months), log-rank (Mantel-Cox) Chi square = 20.7, HR 2.9, 95% CI 1.4–6.0 p<0.0001. **BMI;** body mass index, **CI;** confidence interval, **FAACT A/CS;** Functional Assessment of Anorexia Cachexia Therapy Anorexia/Cachexia Subscale, **HR;** hazard ratio, **OS;** overall survival, **p;** p-value relating to Mantel-Cox Chi square test, **PS;** performance status.

Among patients with metastatic disease, the median OS of those with higher BMI (> 25 kg/m^2^) was longer (11.6 months) than those with BMI≤25 (6.5 months, P = 0.05), as shown in **Fig 4A**. The median OS of patients who had lost less than 5% weight loss in the six months prior to their cancer diagnosis was not significantly different (9.0 months) to that of patients with who had experienced ≥5% weight loss (8.4 months), (HR 0.93, 95% CI 0.56–1.56 P = 0.26), as shown in **Fig 4B**.

The median OS of patients with FAACT A/CS scores of >37 was longer (19.3 months) than patients with scores of ≤37 (6.7 months), (HR 2.9, 95% CI 1.4–6.0 P<0.0001), as shown in **Fig 4C**.

**Fig 5:**
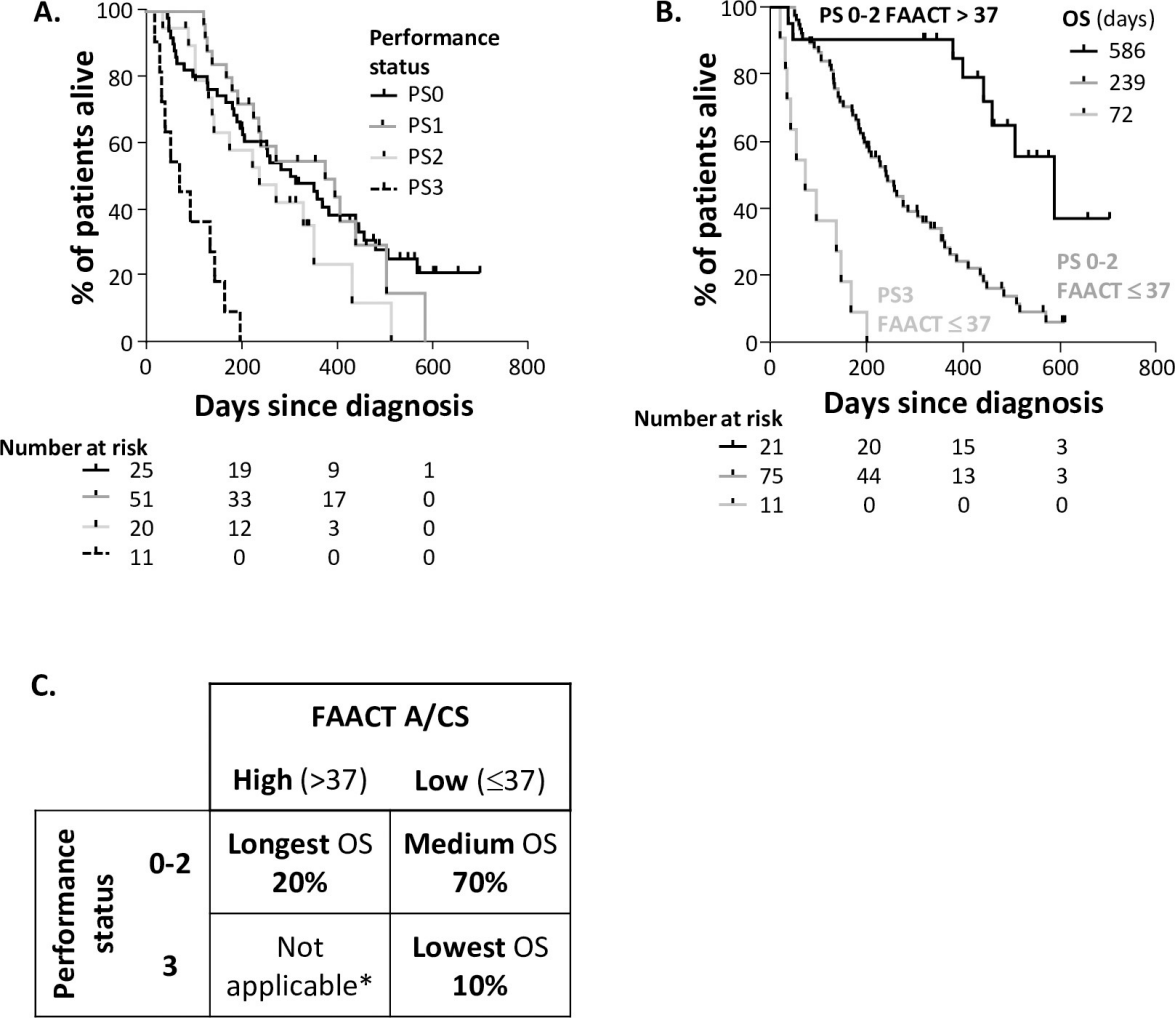
Effect of Performance Status in N = 107 patients with metastatic cancer. **A.** Survival outcomes for patients divided by performance status; the median OS for patients with PS 3 was significantly lower (2.4 months) than this with PS0 (12.4 months), log-rank (Mantel-Cox) Chi square = 30.1, HR 5.2, 95% CI 2.5–11.2 P<0.001. There was little difference observed between PS0 and PS3. **B.** A comparison of survival outcomes for patients divided by PS and FAACT A/CS; between the PS0-2 groups, Log-rank (Mantel-Cox) Chi square = 18.1, P<0.0001, HR 2.5 (95% CI 1.2–5.1). **C.** Summary of survival outcomes in N = 107 patients with metastatic cancer; % shown are the % of N = 107 patients with metastatic disease in this study. *Not applicable because all N = 11 Performance Status 3 patients also had low FAACT A/CS. **BMI;** body mass index, **FAACT A/CS;** Functional Assessment of Anorexia Cachexia Therapy Anorexia/Cachexia Subscale, **OS;** overall survival, **PS;** performance status. P values indicated are t-test (type 2, 2-tailed). N = 14 patients were categorised as ‘unclear’ weight loss over the previous 6 months, so have been removed from all evaluations involving weight loss.

**Fig 6:**
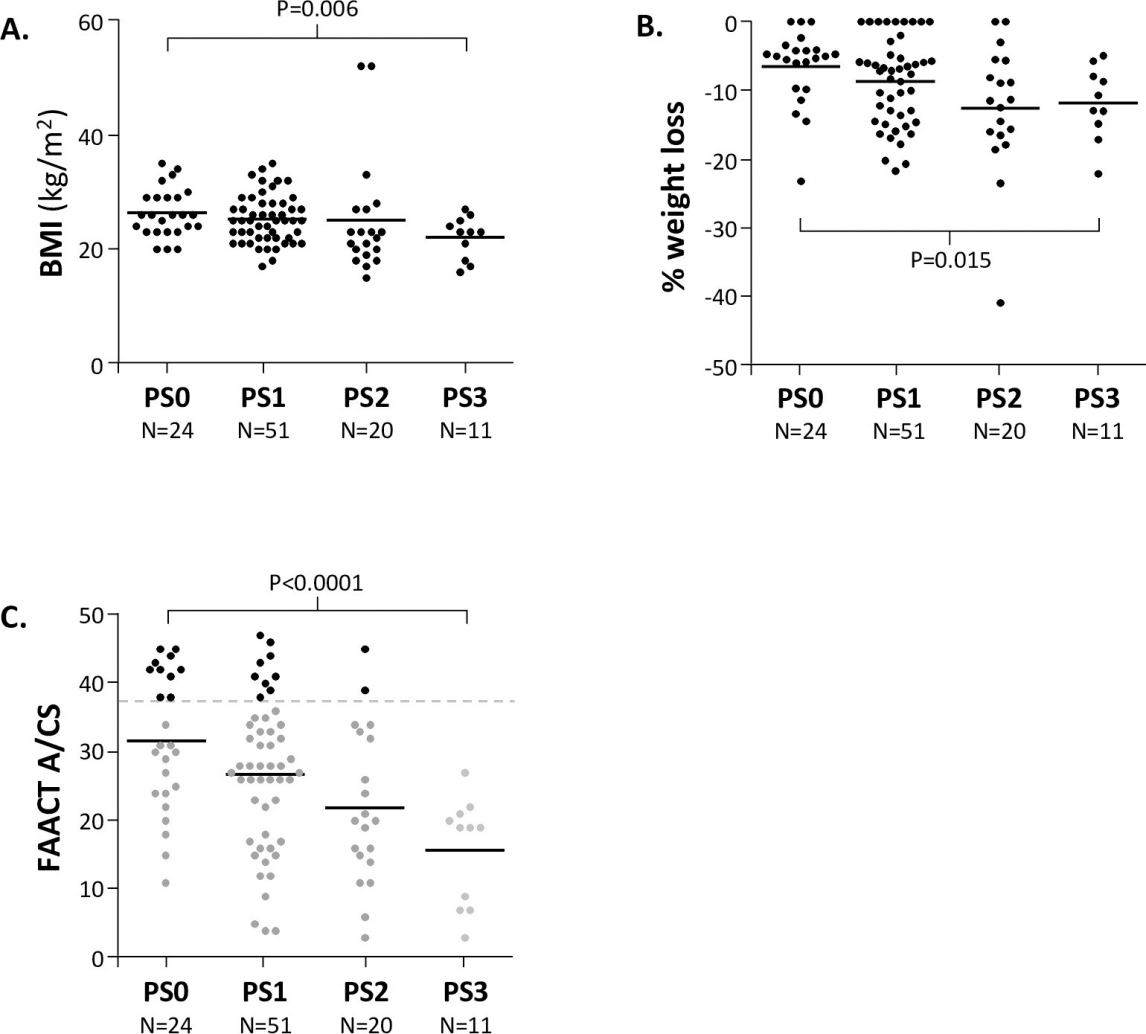
Effect of Performance Status in N = 107 patients with metastatic cancer. **A.** The relationship between PS and BMI, **B.** The relationship between % weight loss and PS, **C.** The relationship between FAACT A/CS and PS; the light grey, dark grey and black dots indicate the three populations of patients in which survival outcomes are compared in **Fig 4B**. **BMI;** body mass index, **FAACT A/CS;** Functional Assessment of Anorexia Cachexia Therapy Anorexia/Cachexia Subscale, **PS;** performance status. P values indicated are t-test (type 2, 2-tailed). N = 14 patients were categorised as ‘unclear’ weight loss over the previous 6 months, so have been removed from all evaluations involving weight loss.

When examining the ECOG PS of patients with metastatic disease, as shown in **Fig 5A**, patients with PS 0, 1 and 2 did not differ in terms of median OS, while only those with PS 3 had significantly shorter median OS. We therefore examined the prognostic role of different parameters within the group of patients with PS 0–2. As shown in **Fig 6,** we observed a significant correlation between PS and BMI, percentage weight loss or FAACT A/CS score. However, a substantial number of PS0-2 patients also had FAACT A/CS scores of ≤ 37 (60% PS0, 82% PS1, 90% PS2).

As shown in **Fig 5B**, the survival outcomes of patients with both PS0-2 and with FAACT A/CS scores > 37 are substantially longer than those with PS 0–2 and FAACT A/CS scores ≤ 37 (HR 2.5, 95% CI 1.2–5.1, P<0.0001). **Fig 5C** summarises the relationship between PS and FAACT A/CS scores. Patients with PS 0–2 and FAACT A/CS scores > 37 had the longest OS outcomes; this represented 20% of metastatic patients. Patients with PS3 and FAACT A/CS scores ≤ 37 had the shortest OS outcomes; 10% of metastatic patients (NB there were no PS3 patients with FAACT A/CS scores >37). Patients with PS0-2 and FAACT A/CS scores ≤ 37 had average OS outcomes; this represented 70% of the metastatic patients and is likely to define those patients who may benefit from early nutrition intervention.

**Table 3** shows the univariate and multivariable Cox regression analyses including patients with metastatic cancer. In the univariate analysis, gender did not impact on clinical outcome, despite the fact that 85% patients with oesophageal/GOJ cancer and 59% of patients with gastric cancer were men. Similarly age, BMI and O’Rourke dysphagia score were not associated with outcome.

**Table 3:** Univariate and multivariate Cox Regressions for patients with metastatic disease.

	Univariate Cox Regression			Multivariable Cox Regression for N = 30 untreated patients			Multivariable Cox Regression for N = 77 treated patients		
	P value	HR	95% CI	P value	HR	95% CI	P value	HR	95% CI
**Gender** (female, male)	0.082	1.62	0.94–2.78						
**Age**	0.847	1.00	0.98–1.02						
**O'Rourke dysphagia score**	0.122	1.21	0.95–1.55						
**Treatment**	**<0.0001**	**0.17**	**0.10–0.27**						
**Performance status** (PS)	**<0.0001**	** **		0.122			0.236		
PS 0*	**1**	** **		1			1		
PS 1	0.985	0.99	0.56–1.78	0.76	0.72	0.09–6.07	0.092	0.56	0.28–1.01
PS 2	0.190	1.60	0.79–3.22	0.25	0.27	0.03–2.50	0.565	0.75	0.29–1.98
PS 3	**<0.0001**	**7.84**	**3.47–17.68**	0.94	1.09	0.13–8.75			
**Total number of metastases**	0.311	1.14	0.89–1.45						
Liver metastases yes vs no	0.266	1.29	0.82–2.01						
Peritoneal metastases yes vs no	0.734	1.10	0.63–1.91						
Other metastases yes vs no	**0.041**	**1.96**	**1.03–3.75**	0.82	0.85	0.21–3.48	0.227	1.64	0.74–3.63
**BMI** (≤ 25 vs >25 kg/m^2^)	0.052	0.64	0.41–1.00	**0.02**	**5.61**	**1.86–16.96**	0.870	1.06	0.54–2.10
FAACT AC/S total**	**<0.0001**	**0.67**	**0.55–0.81**	0.324	0.76	0.43–1.32	**0.016**	**0.70**	**0.53–0.94**

*PS0 was used as a reference.

**HR per 10 units of FAACT AC/S change. Patients defined as ‘untreated’ did not receive active treatment for their cancer, whereas patients defined as ‘treated’ did receive active cancer treatment.

Metastatic sites were categorised as liver, peritoneal or other (adrenal, bone, lung, nodes, omentum, ovary, renal, retrothyroid, pleura, skin).

Cancer treatment substantially improved OS (HR 0.17, 95% CI 0.10–0.27, P<0.0001). When PS 0 was used as reference value for survival analysis, only PS 3 showed a significant association with poor prognosis as previously reflected in Kaplan-Meier curves. Neither the total number of metastatic sites, nor the presence specifically of liver or peritoneal metastases was associated with worse outcomes. However ‘other’ sites of metastases were grouped together (due to smaller numbers in each sub-group), which included adrenal, bone, lung, nodes, omentum, ovary, renal, retrothyroid, pleura and skin metastases; this group were associated with poorer survival outcomes. Finally, FAACT A/CS scores of ≤ 37 were associated with shorter survival outcomes. In the multivariate Cox regression, only BMI was associated with shorter OS in the N = 30 patients who did not receive cancer treatment. Conversely, only FAACT A/CS scores were associated with shorter OS (**Table 3**). Of the 77 patients with metastatic disease who started chemotherapy, 58 patients had a CT scan after 3 cycles. Of these, 51 patients had either a partial response or stable disease; 19 had FAACT A/CS ≤37 and 32 had FAACT A/CS > 37. The median OS for each group was 586 and 408 days respectively, P = 0.037, HR 1.5 (95% CI 0.57–3.6) which suggests the FAACT A/CS is providing information additional to treatment response.

**Fig 7:**
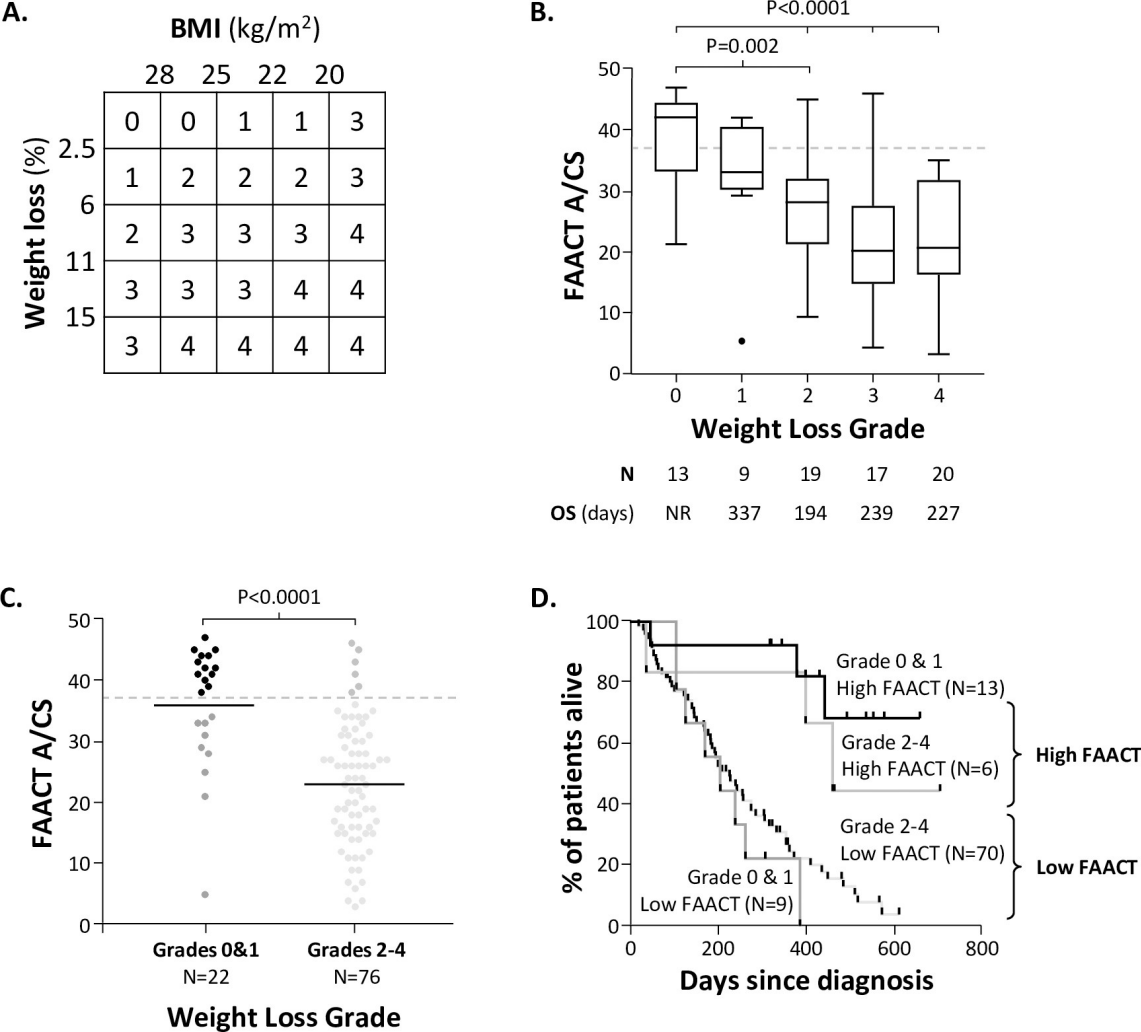
BMI-adjusted weight loss grading system for patients with cancer cachexia. A weight loss grading system was devised^9^ which combines weight loss with BMI to produce a grade from 0 to 4; in two large studies^9,10^ higher weight loss grades have resulted in poorer survival outcomes. This weight loss grading system was applied to the N = 98 patients with metastatic cancer for whom we also have data for % weight loss in the 6 months prior to diagnosis. **B.** Shows the FAACT A/CS scores by weight loss grade. Weight loss grade 1–4 was not discriminatory for OS in this study (which may be due to the small number of patients in some categories. **C.** Shows the relationship between FAACT A/CS scores and weight loss grade (grouped by 0 & 1 and 2–4); the black, dark grey, mid-grey and light grey colours indicate the four populations of patients used in D, **D.** A comparison of survival outcomes for patients divided by weight loss grade and FAACT A/CS; this shows that FAACT A/CS scores are a more discriminatory tool compared with BMI-adjusted weight loss grade (which is related to OS, but only as a big population). Box and whisker plots show the median, upper and lower quartiles and max/min (excluding outliers), P values indicated are t-test (type 2, 2-tailed). N = 9 patients with metastatic cancers were categorised as ‘unclear’ weight loss over the previous 6 months, so have been removed from all evaluations involving weight loss. **BMI;** body mass index, **FAACT A/CS;** Functional Assessment of Anorexia Cachexia Therapy Anorexia/Cachexia Subscale, **OS;** overall survival, **PS;** performance status.

A BMI-adjusted weight loss grading system for patients with cancer cachexia has been devised [14] which combines weight loss with BMI to produce a grade from 0 to 4 (summarised in **Fig 7A**); in two large studies^(15,16)^ higher weight loss grades have resulted in poorer survival outcomes. This weight loss grading system was applied to the N = 98 patients with metastatic cancer for whom we also had data for % weight loss in the 6 months prior to diagnosis.

**Fig 7B** shows the FAACT A/CS scored by weight loss grade. FAACT A/CS scores were highest in patients with weight loss grade 0, but weight loss grades 1–4 had overlapping FAACT A/CS scores and were not discriminatory for OS in this study (which may be due to the small number of patients in some categories).

**Fig 7C** shows patients grouped into either weight loss grade 0–1 or 2–4 and then compares FAACT A/CS scores; these two groups of patients have significantly different mean FAACT A/CS values. When the survival outcomes of these four groups of patients were compared (**Fig 7D**), FAACT A/CS is shown to be a more discriminatory tool compared with BMI-adjusted weight loss grade.

## Discussion

The survival outcomes for patients with OG cancer, both in the curative and non-curative setting remain very poor. Five-year survival rates range from 4% to 30% for patients with non-resectable [16] and resectable disease, respectively [17]. Attempts to improve these outcomes have focused mainly on developing new anti-cancer therapies; there has been less consideration for other factors which may also influence survival outcome.

In recent years, interest in how quality of life measures influence survival has grown. In this study, we hypothesised that anorexia, diagnosed based on the FAACT A/CS, was a more effective tool than either BMI or weight change in providing information about the process of deterioration in nutritional pathology and association with survival outcomes. This study shows that anorexia affects 69% patients with OG cancer, which represents an important component contributing to reducing quality of life for some of these patients [8]. It is interesting to note that anorexia was already present in the non-metastatic state, a finding also observed by Muscaritoli and colleagues [18].

The median OS of metastatic patients with FAACT A/CS scores of >37 was significantly longer than patients with scores of ≤37 (19.3 months vs 6.7 months, HR 2.9, P<0.0001). When combining anorexia score and PS, patients with PS 0–2 and FAACT A/CS >37 had substantially longer OS than those with PS 0–2 and FAACT A/CS ≤ 37 (18.7 months vs 7.9 months, HR 2.5, P<0.0001). Importantly, this is the first study we are aware of that demonstrated anorexia is a prognostic factor in patients with metastatic OG cancer.

In clinical practice, it is important to recognise anorexia may be the initiating trigger that cascades to activate other nutritional pathologies, which in turn lead to a general decline in a patient’s wellbeing. When anorexia develops, there is often a reduction in food intake; in combination with abnormal metabolism, cachexia ensues. Although a working definition of cachexia exists [1], measures of one of its components, anorexia, is often not collected in the clinic setting. Therefore, cachexia is often only identified beyond the pre-cachectic state, where it cannot be fully reversed by nutrition support alone. Refractory cachexia is irreversible and often regarded as a pre-terminal state, associated with end of life. However, the pre-cachexia stage is potentially reversible and in the absence of any validated biomarkers for cachexia [11] identifying anorexia could be used to detect the cachexia process earlier and, hence, treat it in the reversible pre-cachexia stage (less than 5% weight loss in the 6 months prior to cancer diagnosis).

In general oncology practice, BMI and alterations in weight are used as surrogate measures of the processes described above. We used the FAACTS A/CS to assess for anorexia in patients diagnosed with upper GI cancers, who may have experienced anorexia prior to their cancer diagnosis. The 12 questions forming the subscale were found to be a better predictor of OS than either BMI or weight loss.

BMI was only an informative metric in patients who did not receive treatment for their cancer. This observation is supported by other studies; for example Ock *et al* concluded that BMI was less useful as a tool for predicting clinical outcome in 719 patients receiving palliative chemotherapy for advanced gastric cancer [6]. Instead, it has been proposed that the use of body composition to identify sarcopenia has the potential to become a clinically significant tool to support decision making in patients with OG cancer. Multiple studies have demonstrated sarcopenia can result in treatment delays through poor tolerance to treatments and may also, independently, be a predictor of poor survival 19,20]. However, the lack of standardisation of methods for assessing and reporting body composition in this patient group limits assessment [6,7]. This is an area of ongoing research.

Weight loss is routinely and easily measured in oncology clinics. In our study, patients with metastatic cancer had a greater six-month pre-cancer diagnosis percentage weight loss than those with non-metastatic cancer. However, in patients with metastatic cancer there was no statistical difference in survival outcomes between patients who had lost <5% and ≥5% weight in the six months prior to cancer diagnosis. A possible explanation for this is that although it is accepted that identification of weight loss pre-treatment is paramount, especially in patients that continue to lose weight, it is less clear what the effects of pre-treatment weight loss are on survival if the weight loss is aggressively corrected during treatment. For example, weight may be gained by increasing calorific intake without improving the anorectic state or a patient may put weight on if a clinical/ radiological response (including improvement in anorectic state) to systemic chemotherapy is achieved. Studies have shown that even patients with initial weight loss can recover weight and experience an improved prognosis if they receive appropriate treatment, e.g. nutrition interventions. Patients who experience early weight loss and continue to lose weight have the poorest OS [18,19].

The negative effects of weight loss on cancer patients has long been known; a study in 1980 reported that 29% of patients with measurable gastric cancer reported 5–10% weight loss in the previous 6 months and 38% of patients reported more than 10% weight loss, which together represented two thirds of these patients [20]. A large retrospective study of N = 1555 patients with GI cancers of the oesophagus, stomach, pancreas, colon or rectum who were to receive chemotherapy, reported that weight loss at presentation was more common in men (51%) than women (44%). Patients who had experienced weight loss received lower chemotherapy doses initially and developed more frequent and more severe dose limiting toxicity (plantar-palmar syndrome and stomatitis) and received 1 month (18%) less treatment. However, it is challenging to comprehend fully the quantity and significance of weight loss pre-diagnosis as it is difficult to ascertain the time at which the pre-cancerous individual develops cancer and patient recall bias is also possible [5]. In a study investigating pre-diagnosis weight loss in N = 134 patients with early-stage oesophageal cancer, median weight loss was 4.7% (inter-quartile range 0%-10.9%). BMI one year prior to diagnosis was not associated with mortality. Pre-diagnosis weight loss was associated with increased all-cause mortality, but only in patients with early stage oesophageal cancer [21].

It is important to understand how anorexia associates itself with weight loss to influence survival. In our study, out of the 107 metastatic patients for whom there was survival data, we examined the 98 patients (87%) who had registered a FAACT A/ CS score of ≤37 and found that there was no statistical difference in survival between those patients who lost ≥5% weight and those that had not. This suggests anorexia as diagnosed by the FAACT A/CS score is independent of weight loss as a predictor of survival. Similarly, in the same cohort of patients, we also looked at the relationship between treatment response and FAACT AC/S score. We looked to see if radiological response to therapy affected how informative the FAACT AC/S score was.

### Moving forward

Nutritional status affects acceptability and tolerability of anticancer therapies, in turn altering therapeutic choices [17]. An accurate assessment of nutritional status is of paramount importance in treating cancer patients, and anorexia should be considered as part of this assessment.

The European School of Oncology Task Force position paper [10] and the European Society for Clinical Nutrition and Metabolism (ESPEN) guidelines on nutrition in patients with cancer both advocate moving the focus from end-stage wasting, to supporting patients' nutritional and functional state throughout the increasingly complex and prolonged anti-cancer treatment phase. This should include both ensuring sufficient energy intake and maintaining physical activity to maintain muscle mass, along with reducing systemic inflammation (if required). However, management of the latter remains challenging as although c-reactive protein (CRP) is the most robust biomarker to identify cachexia inflammation, it is neither specific for cancer, cachexia or for tumour activity as it can be influenced by other factors such as infections [22].

However, these approaches do not advise assessing for anorexia, which may predispose to cachexia and should be seen as a separate pathology. There are numerous factors that contribute to the development of anorexia and each factor should be addressed individually; a ‘one-size-fits-all’ approach is unlikely to be effective. Recent phase 3 trials using pharmaceutical agents targeting cachexia in non-small cell lung cancer patients [23] showed improvement in appetite scores using the FAACT A/CS although the intervention drug failed American Food and Drug Administration approval.

In this way, earlier screening for anorexia with subsequent nutrition intervention (with or without therapeutic agents that target appetite) if appropriate, may enhance the quality of life of patients with advanced cancers. In addition this may also provide an opportunity to optimise response to anticancer treatments. In clinical practice, it is important to have easily applicable measurements/assessments, and the use of the FAACT A/CS shows anorexia can be measured objectively and used to identify patients suitable for early nutritional interventions in the patient journey.

## Main conclusions from this study

This study has demonstrated that FAACT A/CS questionnaire, a simple set of 12 multiple choice questions, can be administered in a real-world clinical setting and can used as an effective predictor of survival. Results can be interpreted without specific expertise and do not rely on patients being sufficiently informed to describe anorexia if they are unaware of this condition. Over two thirds (69%) of all patients are considered to be anorexic using the FAACT A/CS. This questionnaire was a better predictor of OS than BMI or weight loss. It is important for clinicians to acknowledge that only 62% of anorexic patients reported significant weight loss in the six months prior to cancer diagnosis, indicating that anorexia may be independent of weight loss. Therefore anorexia should be addressed as a separate pathology. Impact of PS on OS highlights the importance of combined use of PS and FAACT A/CS to identify groups of patients who may benefit from early and targeted nutrition interventions. Effective nutritional intervention can only be achieved if there is a thorough assessment of weight loss history, eating behaviour, changes in appetite, and the presence of nutrition impact symptoms. In order to improve patient outcomes, the next steps should include investigations into change in FAACT A/CS values during treatment and interventions to see if scores can be proactively improved with any impact on survival. This is an area of unmet need, which is likely to affect patients with cancers other than oesophagogastric cancers.
